# Investigating dynamics, etiology, pathology, and therapeutic interventions of *Caligus clemensi* and *Vibrio alginolyticus* co-infection in farmed marine fish

**DOI:** 10.1038/s41598-024-70528-x

**Published:** 2024-09-05

**Authors:** Mohamed Abdelsalam, Marwa M. Attia, Mohamed Sayed Marzouk, Reda M. S. Korany, Mamdouh Y. Elgendy, Asmaa W. Soliman, Abdelbary Prince, Ahmed H. Hamada

**Affiliations:** 1 Department of Aquatic Animal Medicine and Management, Faculty of Veterinary Medicine, Cairo University, PO 12211, Giza, Egypt; 2 Department of Parasitology, Faculty of Veterinary Medicine, Cairo University, PO 12211, Giza, Egypt; 3 Department of Pathology, Faculty of Veterinary Medicine, Cairo University, 12211, Giza, Egypt; 4 Hydrobiology Department, Veterinary Research Institute, National Research Centre, Dokki, 12622, Cairo, Egypt; 5 National Institute of Oceanography and Fisheries (NIOF), Cairo, Egypt; 6 Department of Biochemistry and Molecular Biology, Faculty of Veterinary Medicine, Cairo University, 12211, Giza, Egypt; 7 Department of Fish Production, National Company for Fisheries and Aquaculture, Ministry of Defense, Cairo, Egypt

**Keywords:** *Caligus clemensi*, *Vibrio alginolyticus*, European seabass, Flathead grey mullet, Aquaculture, Secondary infections, Nature-identical compound, Probiotics, Biological techniques, Microbiology

## Abstract

This study investigated a disease outbreak characterized by caligid copepod infestations and subsequent secondary bacterial infections in European seabass (*Dicentrarchus labrax*) and flathead grey mullet (*Mugil cephalus*) cultivated at a private facility in the Deeba Triangle region of Egypt. Moribund fish displayed brown spots on the skin, tongue, and gills, along with lethargy and excess mucus. The fish suffered severe infections, exhibiting external hemorrhages, ulcers, and ascites. The fish had pale, enlarged livers with hemorrhaging. Comprehensive parasitological, bacteriological, molecular, immunity and histopathological analyses were conducted to identify the etiological agents and pathological changes. Caligid copepod infestation was observed in wet mounts from the buccal and branchial cavities of all examined fish, and the caligids were identified as *Caligus clemensi* through *COI* gene sequencing and phylogenetic analysis. *Vibrio alginolyticus* was confirmed as a secondary bacterial infection through biochemical tests, *recA* gene sequencing, and phylogenetic analyses. Antibiotic susceptibility testing revealed resistance to β-lactams, aminoglycosides, and trimethoprim-sulfamethoxazole in *V. alginolyticus* isolates. Upregulation of the inflammatory marker *IL-1β* in gill and skin tissues indicated a robust cell-mediated immune response against the pathogens. Histopathological examination revealed severe tissue damage, hyperplasia, hemorrhage, and congestion in the gills, along with hepatocellular degeneration and steatosis in the liver, providing initial insights into this outbreak. A comprehensive therapeutic regimen was implemented, comprising prolonged hydrogen peroxide immersion baths, followed by the application of the nature-identical plant-based compound Lice-less and probiotic Sanolife Pro-W supplementation. This integrated approach effectively eliminated *C. clemensi* infestations, controlled secondary bacterial infections, and restored fish health, reducing morbidity and mortality rates to minimal levels.

## Introduction

The mariculture industry in Egypt, particularly in the Deeba Triangle region along the Mediterranean coast, significantly contributes to commercial marine fish production^1,2^. The Deeba Triangle, influenced by both the Mediterranean Sea and nearby Lake Manzalla, experiences a dynamic interplay of tidal forces and seasonal fluctuations in water parameters^3^. This dynamic environment can directly impact the physiology of cultivated fish species^4^. The European seabass (*Dicentrarchus labrax* L.), belonging to the Moronidae family, is the dominant mariculture species in Egypt, contributing approximately 13.4% of the global farmed seabass production, following Turkey and Greece^5^. Well-adapted to mariculture, *D. labrax* tolerates a wide range of salinities and temperatures^6^. Grey mullet (*Mugil cephalus*) farming also plays a crucial role, with Egypt accounting for over 60% of Africa’s total mullet culture and contributing over 12% of the country’s annual fish production^7^. However, parasitic crustaceans and vibriosis pose significant threats to seabass and mullet mariculture, causing mortality and economic losses, especially in suboptimal conditions^8^.

One such parasitic group, the caligid copepods or sea lice, has a widespread distribution and a detrimental impact on various marine and brackish water fish species^9^. The family Caligidae comprises numerous genera and species, with the genus *Caligus* being the largest, encompassing 267 recognized species and exhibiting morphological variations^10^. Accurate identification and differentiation within this genus have been hindered by limited knowledge, prompting the use of molecular techniques like *COI* gene sequencing to confirm taxonomic distinctions^11^. The prevalence and life cycles of *Caligus* spp. are intrinsically linked to marine ecosystem dynamics, shaped by factors such as sea temperature fluctuations, food availability, and the feeding strategies adopted by their larval stages, which can be either planktotrophic or lecithotrophic^12^. Egg-laying species like C*aligus clemensi*, *C. elongatus*, *C. rogercresseyi*, and *Lepeophtheirus salmonis* exhibit year-round female presence, with some capable of hatching and advancing to the infective stage even at low temperatures^13^. Rising temperatures contribute to an increased prevalence of sea lice infestations, and rapid temperature fluctuations can heighten fish susceptibility to diseases^14^.

Sea lice infestations pose a significant challenge to marine fish farming globally, with reports from various countries, including Egypt. The feeding behavior of *Caligus* spp., involving the consumption of host mucus and blood, leads to skin erosion, ulceration, and potential secondary infections, ultimately compromising osmoregulation and overall fish health^9^. Caligid copepods have been identified as potential vectors for transmitting secondary bacterial and viral infections, including vibriosis^8^. Bacterial pathogens were found in the midgut of salmon sea lice, suggesting a possible link to disease transmission^15^. *Vibrio alginolyticus* has been found in *C. lalandei* and *C. minimus*, posing risks to aquaculture operations and human health^16^. Sea lice infestations and associated secondary bacterial infections pose economic threats to mariculture. Conventional control measures employing organophosphates and antibiotics raise environmental concerns and promote resistance development^17^. Consequently, eco-friendly alternatives are urgently needed. The blend of nature-identical compounds carvacrol, thymol, and cinnamaldehyde exhibits promising antiparasitic, antimicrobial, immunomodulatory, and growth-promoting effects in fish^18^. Concurrently, hydrogen peroxide and probiotics have shown potential in controlling sea lice and bacterial pathogens^9^.

Elevated mortality rates in semi-intensive seabass and mullet mariculture in the Deeba Triangle region have been attributed to caligid copepod infestations and secondary bacterial infection. This multifaceted study aimed to identify the specific causative agents through comprehensive morphological and molecular analyses, elucidate host–pathogen interactions via histopathological examinations, and investigate the expression of interleukin-1β (*IL-1β*). Additionally, the efficacy of a customized control program incorporating the nature-identical compound blend, hydrogen peroxide, and probiotics was evaluated to support the development of sustainable and eco-friendly strategies for managing co-infections in mariculture.

## Materials and methods

### Ethical approval

This study’ methods and trial protocols complied with the relevant guidelines and regulations for the use of fish in research, as stipulated by the American Fisheries Society (AFS, 2014). Approval was granted by the Ethics Committee of the Faculty of Veterinary Medicine at Cairo University, Giza, Egypt (VET-CU-IACUC-03162022629).

### Farm investigation

This study was conducted in the Deeba Triangle region (31°24′46″N 31°52′38″E), bounded by the Mediterranean Sea to the north, the Estuary of Damietta to the west, and Lake Manzalla to the south. This study was initiated in response to significant mortality among farmed European seabass and grey mullet at a private fish farm (El-Deepa Farm) in this region. No previous history of parasitic or bacterial diseases had been recorded among farmed fish in this particular farm, making this outbreak notable and warranting investigation. The study spanned three months (February, March, and April 2022), during which regular visits to the affected farm facilitated the evaluation of mortality rates, clinical manifestations, biosecurity measures, and the application of integrated therapeutic interventions to control the observed morbidity and mortality. The farm's aquaculture practices included stocking European seabass seeds from hatcheries into separate earthen ponds at a density of approximately 6000 fish per pond in monoculture systems. In contrast, grey mullet fries were wild-caught and then stocked in the farm. These grey mullets were either cultivated in monoculture ponds or polycultured with seabream. The farm consisted of several 6900 m^3^ earthen ponds with a water depth of 1.7 m. Fish were fed with 38% crude protein sinking pellets (Aller, Egypt). Initial investigations revealed that caligid parasitic copepod infestations were leading to secondary bacterial infections, which likely contributed to the mortality incidents across the ponds. This co-infection scenario presented a complex challenge that required a comprehensive approach to diagnosis and treatment.

### Fish sampling

A total of 800 diseased fish samples were randomly collected from the outbreak farm in the Deeba region during February and March 2022, including 400 European sea bass (*D. labrax*) and 400 flathead grey mullet (*M. cephalus*). The fish specimens ranged in total body weight from 290 to 320 g and total length from 29 to 32 cm for seabass, while for mullet, the weight ranged from 140 to 170 g and total length from 21 to 24 cm. The collected specimens were promptly transported to the wet laboratory using isothermal containers filled with crushed ice. Clinical, post-mortem and histopathological examinations were performed in the laboratory, along with parasitological, bacteriological, and molecular investigations^19^.

### Parasitological investigation

Each fish underwent necropsy, with the gills and skin dissected and scraped for examination under a dissecting microscope. The copepod were cleared using lactophenol and mounted in glycerin jelly. Multiple criteria were carefully assessed for identifying the mounted Copepoda, including counting the segments of antennae and antennules, calculating the length-to-width ratio, evaluating the number of setae on each segment, and measuring the length of the egg sac^20^. Measurements were recorded in micrometers as the mean value ± standard deviation (SD). The copepod specimens were mounted and illustrated using a camera lucida apparatus connected to an Olympus BH2 microscope^21^. The identification procedure followed the taxonomic methodology described by El-Rashidy and Boxshall^22^. The prevalence, and mean intensity of caligid copepod parasites infesting seabass and grey mullet were carefully determined**.**

### Electron microscopy analysis

Scanning electron microscopy was employed to analyze the ultra-morphological features of the caligid copepod parasites. The parasites were preserved using a 2.5% glutaraldehyde solution and dehydrated through a sequential series of ethanol concentrations (50, 70, 90, and 100%) for 10 min per concentration. The whole parasitic copepod specimen underwent dehydration using a CO_2_ critical point drier (Autosamdri-815, Germany). The copepods were mounted onto scanning electron microscope stubs, coated with a 20 nm layer of gold using a sputter coater (Spi-Module sputter coater, UK), and imaged using a JEOL JSM 5200 electron probe microanalyzer at the Faculty of Agriculture, Cairo University, Egypt. All measurements are expressed in millimeters.

### Sequencing of *COI* gene

Genomic DNA was isolated and purified from individual and pooled adult copepod specimens using the DNeasy Tissue Kit following the manufacturer's protocol for animal tissues (QIAGEN Inc., Mississauga, Ontario, Canada). The extracted DNA was quantified and quality-checked using a NanoDrop spectrophotometer (Thermo Scientific NANODROP 2000), then stored at − 20 °C until further use.

The mitochondrial cytochrome c oxidase I (*COI*) gene was PCR amplified using the primer pair LCO1490 (5′-GGT CAA CAA ATC ATA AAG ATA TTG G-3′) and HCO2198 (5′-TAA ACT TCA GGG TGA CCA AAA AAT CA-3′)^23^. PCR amplification was performed using GoTaq Polymerase (Promega, Madison, WI, USA). The PCR thermal cycling conditions included an initial denaturation step at 94 °C for 5 min, followed by 40 cycles of denaturation at 94 °C for 1 min, annealing at 40 °C for 90 s, and extension at 72 °C for 2 min^24^. The amplified PCR products were visualized by agarose gel electrophoresis. Amplicons of the expected size were excised from the gel and purified using the QIAquick Gel Extraction Kit (QIAGEN, Hilden, Germany). The purified amplicons were sent for Sanger sequencing at Macrogen Inc. (Seoul, Korea).

The obtained *COI* gene sequences were assembled and edited using BioEdit software^25^, and subsequently deposited in GenBank. The current *COI* sequences were compared with 28 other Caligidae accessions, which showed more than 87% similarity to the current *Caligus* sp. Multiple sequence alignment and phylogenetic tree reconstruction were performed using the maximum likelihood methodology in MEGA11 software^26^**,** employing the GTR + G + I model of nucleotide substitution. This model was selected based on model selection criteria to provide optimal accuracy for the dataset. *Nemesis lamna* (KF931070) was used as the outgroup taxon.

### Bacteriological examination

Liver and kidney samples from fish were aseptically homogenized in 1 ml of sterile saline solution. Subsequently, aliquots of 100 μl from the homogenates were inoculated onto marine agar plates (Difco, USA) and thiosulfate citrate bile salts sucrose (TCBS) agar (Oxoid™), and plates were incubated overnight at 30 °C. The selected colonies were purified, and subjected to phenotypic identification procedures, including Gram staining, growth on TCBS agar, sensitivity to the vibriostatic agent O/129 (2,4-diamino-6,7-diisopropylpteridine phosphate) disc, and biochemical characterization using the API 20E identification system. The API-20E system provides a reliable means for identifying frequently encountered species within the Vibrionaceae family^27^. The confirmed isolates were preserved in tryptic soy broth (TSB, Difco, Detroit, MI, USA) supplemented with glycerol at − 80 °C for long-term storage.

### Molecular identification of *Vibrio* sp.

The suspected 8 *Vibrio* isolates were revived from frozen stocks and cultured on tryptone soy agar supplemented with 2% NaCl at 29 °C for 18 h. Genomic DNA was extracted using the Gene Jet Genomic DNA Purification Kit (Thermo Fisher Scientific; Catalog # K0721), following the manufacturer's instructions. The extracted DNA samples were quantified, and their purity was assessed using a NanoDrop2000 spectrophotometer (Thermo Scientific). The purified genomic DNA was stored at -20 °C until further use.

The *recA* gene was amplified by PCR using the *Vibrio* species-specific primer pair recA-01-F (5′-TGARAARCARTTYGGTAAAGG-3′) and recA-02-R (5′-TCRCCNTTRTAGCTRTACC-3′)^28^. The PCR reactions were performed using Dream Taq Green PCR Master Mix (Thermo Fisher Scientific, Waltham, MA). The thermal cycling conditions were as follows: an initial denaturation step at 95 °C for 4 min, followed by 30 amplification cycles consisting of denaturation at 95 °C for 1 min, annealing at 56 °C for 1 min, and elongation at 72 °C for 1 min, with a final extension step at 72 °C for 10 min. The amplified PCR products were purified using a PCR clean-up kit (BioFlux).

The purified *recA* amplicons were sequenced bidirectionally using Sanger sequencing (Macrogene Inc.) with the BigDye Terminator v3.1 kit (Applied Biosystems). The obtained sequence reads were assembled, edited, and analyzed using BioEdit software^25^. The assembled sequences were compared against the GenBank database using BLASTn for species identification and subsequently deposited in the GenBank database under unique accession numbers. A phylogenetic tree was constructed using the maximum likelihood method in MEGA 11^26^. *Salinivibrio costicola* subsp. *costicola* LMG:11651T (AJ842367) was used as an outgroup.

### Antimicrobial susceptibility testing

Eight identified* Vibrio* isolates were cultured in tryptic soy broth (TSB, Difco, Detroit, MI, USA)) supplemented with 2% NaCl at 30 °C for 24 h. The cultures were adjusted to a standardized turbidity of 0.6 at 610 nm using phosphate-buffered saline (PBS, pH 7.2). Mueller–Hinton agar plates (Difco, Sparks, Maryland, USA) supplemented with 1% NaCl were inoculated with 0.1 mL of bacterial suspensions. Available antibiotic disks containing ampicillin (10 µg), amoxicillin (30 μg), gentamicin (10 μg), trimethoprim/sulfamethoxazole (1.25/23.75 µg), florfenicol (30 µg), ciprofloxacin (5 µg), oxytetracycline (30 µg), and novobiocin (30 µg) (Oxoid™, UK) were placed onto the agar surface. The plates were incubated for 24 h at 30 °C. Bacterial inhibition zones were measured, and the susceptibility of each isolate was categorized as susceptible (S), intermediate (I), or resistant (R)^29^.

### Immune-related gene expression analysis

Total RNA was extracted from tissue samples (skin and gills) using the RNeasy mini extraction kit (Qiagen), following the manufacturer's instructions. The immunological experiments were conducted directly on the co-infected fish samples. The concentration and purity of the samples were assessed using spectrophotometry at 260 nm to select pure samples. After removing DNA contamination using DNase I (Fermentas, Lithuania), complementary DNA (cDNA) was synthesized using the Revert Aid First Strand cDNA Synthesis Kit (Thermo-scientific, MA, USA) according to the manufacturer's instructions. For the flathead grey mullet, the primers used were *IL-1β* forward: GAGGAGCTTGGTGCAGAACA and reverse: CTTTGTTCGTCACCTCCTCCA^30^, while for the European seabass, the primer pair F2 (5′-ATCTGGAGGTGGTGGACAAA-3′) and R2 (5′-AGGGTGCTGATGTTCAAACC-3′) was employed to assess the mRNA expression levels of *IL-1β*^31^. Real-time PCR was performed using SYBR Green PCR Master Mix (Thermo-scientific, MA, USA) on the ABI Prism Step One Plus from Applied Biosystems to determine the relative expression of the selected genes. Samples were examined in duplicate. The expression levels of the housekeeping Beta-actin gene were used to normalize the expression levels. The expression levels of *IL-1β* were examined. Genetic expression data were analyzed using the $2^{{ - \Delta \Delta {\text{C}}_{{\text{t}}} }}$ method^32,33^.

### Histopathological examination

Samples from gills and liver were collected from infected fish, fixed in 10% neutral buffered formalin, washed, dehydrated, cleared, and embedded in paraffin. The paraffin-embedded blocks were sectioned at a 5-micron thickness and stained with Hematoxylin and Eosin^34^ for histopathological examination using a light microscope (Olympus BX50, Japan)^35^.

### Field treatment trial

The treatment regimen was implemented in the ponds of the same farm where the co-infection outbreak was identified, and stocking density was maintained. The therapeutic regimen was implemented to include three main successive steps:

1. Initial Treatment: Prolonged immersion baths were performed over two successive days using a potent 40% hydrogen peroxide solution, which was added at a dosage of 4 L/acre during the early mornings. The goal was to disinfect surfaces and contain disease spread within the affected fish. Several p*recA* utions were instituted during the baths. The pond water drainage was closed for 24 h during treatment. Water quality parameters, including dissolved oxygen and pH levels, were carefully monitored. Aerators were operated continuously to maintain oxygenation and optimize H_2_O_2_ efficacy^36,37^.
2. After the two-day H_2_O_2_ regimen, an innovative chemical mixture of commercialized Lice-less (Falcon, Egypt), a locally registered product composed of natural components such as Carvacrol, Thymol, Cinnamaldehyde, Arotec-20, and Vitamin C, was applied for three successive days by mixing with feed (Aller, Egypt) at a ratio of 5 ml/1 kg feed to target the copepod parasites.
3. Subsequently, the probiotic Sanolife Pro-W was applied for an additional two successive days to combat bacterial pathogens. The probiotic was prepared by first adding 100 g to 500 L of water in a holding tank and allowing 4 h of activation before introduction to the ponds^38^.

The overall therapeutic efficacy was evaluated through post-treatment monitoring of mortality rates and comprehensive parasitological and bacteriological testing. Two ponds were left as control groups without application of the therapeutic regimen, to compare the efficacy of treatment. This approach provided insights into the success of hydrogen peroxide disinfectant, Lice-Less, and Sanolife Pro-W probiotic interventions in safeguarding the health and productivity of the farmed fish.

### ARRIVE guidelines

The study was conducted in compliance with the ARRIVE guidelines and all methods were conducted following relevant guidelines and regulations**.**

## Results

### Field observations and disease progression

Initial observations in February 2022 identified infection by the caligid copepod, with morbidity rates of 25 and 15% in European seabass and flathead grey mullet, respectively. Mortality remained low at less than 1% for both species during this initial stage. However, by March, clinical signs indicated progression to co-infection with suspected bacterial pathogens, resulting in a significant escalation of morbidity rates to 65% in seabass and 40% in mullet. Notably, seabream (*Sparus aurata*) polycultured with the infected mullet did not exhibit any clinical signs or infection by *Caligus* sp.

### Clinical signs and postmortem findings

**Fig. 1:**
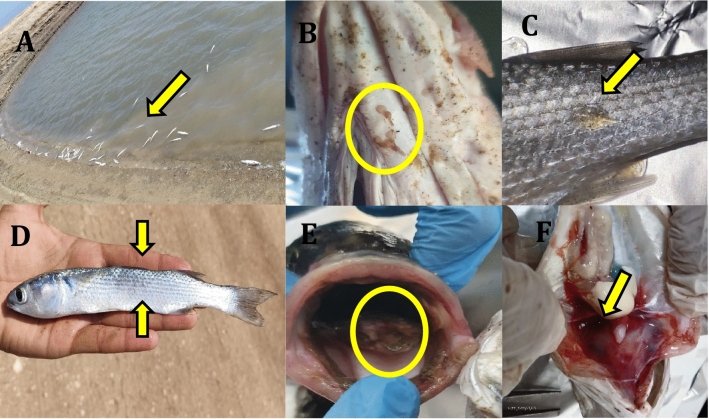
Clinical manifestations in moribund European seabass and grey mullet. (**A**) Several mortalities were observed in semi-intensive ponds stocked with infected European seabass. (**B**) Copepods attached to the lower jaw and isthmus region of an infected seabass. (**C**) Copepods attached to the skin of moribund grey mullet. (**D**) Grey mullet exhibiting emaciation due to infection. (**E**) Ulceration on the tongue of infected European seabass. (**F**) Internal hemorrhages present in the organs of a co-infected European seabass.

Moribund European seabass and flathead grey mullet exhibited common clinical signs, characterized by lethargy and sluggish surface swimming activity. Notably, seabass presented with significant ulceration of the tongue. Clusters of brown spotted dots were observed on the tongue, buccal cavity, and skin of the head and trunk regions in both species. Severely infected fish displayed signs of debilitation, excessive mucus production, gill hemorrhages, loss of appetite, and scale detachment. In the later stages of the disease, infected seabass developed hemorrhagic patches on the gill covers, external ulcers and hemorrhages in the head region, ascites, and ocular opacity. Postmortem examination revealed significant abnormalities, including liver enlargement and paleness. Additionally, hemorrhages and congestion were evident in all internal organs, (Fig. 1).

### Prevalence and intensity of *Caligus* sp. infestation

Our investigation into the dynamics of *Caligus* sp. infestation in farmed marine fish revealed significant patterns in both prevalence and mean intensity (Table 1). In European seabass, the prevalence of infestation increased from 25% in February 2022 to 65% in March 2022, and further to 68% in the untreated group in April 2022. In grey mullet, prevalence increased from 15% in February to 40% in March, and reached 44% in the untreated group in April.

**Table 1:** Prevalence and mean intensity of *Caligus* sp. infestation in European Seabass and grey mullet during outbreak.

Fish species	Season	Examined fish	Patterns of fish infection		Patterns of Caligus intensity	
			Prevalence of Caligus infestation	Prevalence of coinfection (Caligus + Vibrio)	Gills	Skin
European seabass	February 2022	100	25 (25%)	24	6.2 ± 3.2	7.3 ± 1.2
European seabass	March 2022	100	65 (65%)	60	8.2 ± 2.1	9.4 ± 1.6
European seabass (Untreated group)	April 2022	100	68 (68%)	65	9.7 ± 1.1	10.2 ± 1.4
European seabass (Treated group)	April 2022	100	10 (10%)	6	2.1 ± 1.3	1.3 ± 0.2
Grey mullet	February 2022	100	15 (15%)	15	3.2 ± 1.2	4.4 ± 1.5
Grey mullet	March 2022	100	40 (40%)	40	5.1 ± 0.2	7.5 ± 2.3
Grey mullet (untreated group)	April 2022	100	44 (44%)	44	6.3 ± 2.1	8.4 ± 1.7
Grey mullet (Treated group)	April 2022	100	5 (5%)	2	1.2 ± 0.1	1.1 ± 0.3

At the end of May 2022, no morbidity or mortality cases were recorded in treated groups.

The mean intensity of infestation was determined on gills and skin for each infested fish. In February, European seabass showed a mean intensity of 6.2 ± 3.2 parasites on gills and 7.3 ± 1.2 on skin. This increased to 8.2 ± 2.1 on gills and 9.4 ± 1.6 on skin in March, and further to 9.7 ± 1.1 on gills and 10.2 ± 1.4 on skin in the untreated group in April. Grey mullet exhibited mean intensities of 3.2 ± 1.2 on gills and 4.4 ± 1.5 on skin in February, rising to 5.1 ± 0.2 on gills and 7.5 ± 2.3 on skin in March, and reaching 6.3 ± 2.1 on gills and 8.4 ± 1.7 on skin in the untreated group in April. These findings highlight the complex dynamics between *Caligus* sp. and hosts.

### Parasitological examination

**Fig. 2:**
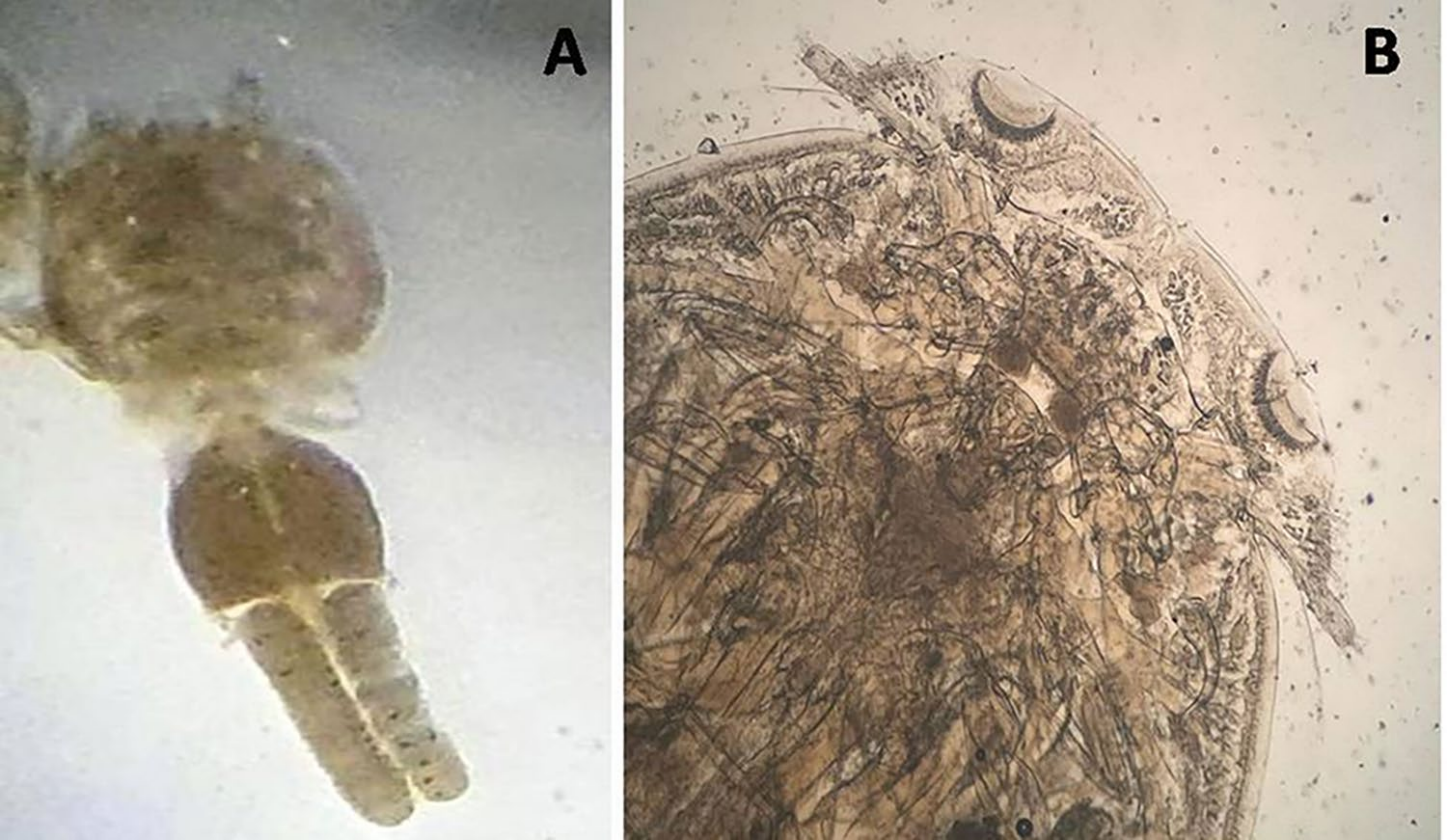
Light microscopic micrograph of adult *C. clemensi*. (**A**) Whole adult which is oval-shaped cephalic, lateral, and thoracic sections comprise the cephalothoracic shield. (**B**) The cephalic area contained a well-developed frontal plate with disk-shaped lunules.

**Fig. 3:**
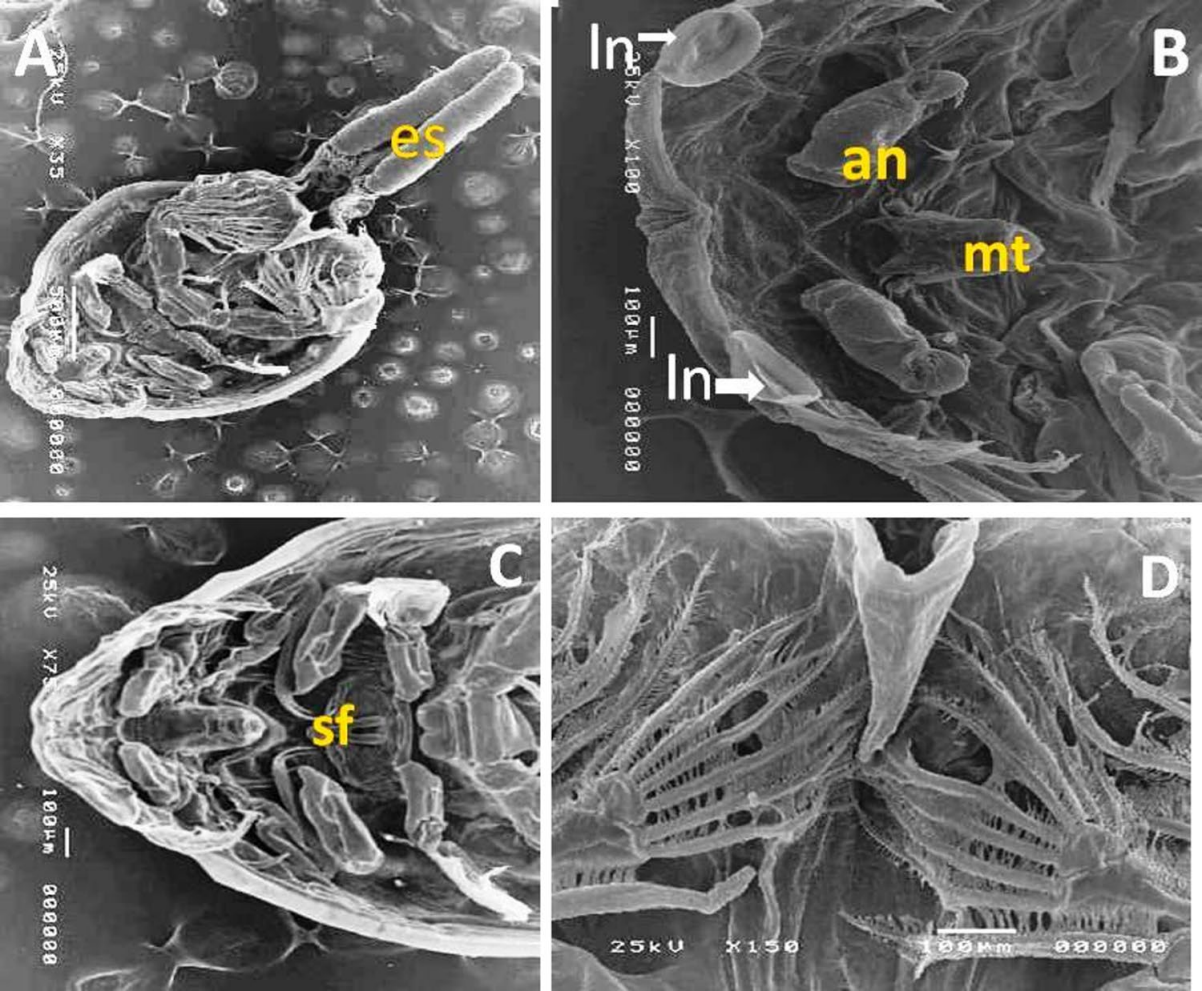
Scanning electron micrograph of adult male and female *C. clemensi* showed: (**A**) Ventral view of adult female caligus with two egg sacs (es); (**B**) Showed the characteristic pair of lunules (ln); segmented antenna (an) and mouth tube (mt); (**C**) Showed the characteristic sternal furca (sf). (**D**) Showed the setae that supported the legs.

Comprehensive parasitological examination of moribund European seabass and flathead mullet specimens revealed a suspected parasitic copepod infestation by *Caligus clemensi*, pending molecular confirmation. These crustacean ectoparasites exhibited distinctive morphological characteristics typical of the genus. The body of *C. clemensi* is segmented, with the number of genital segments ranging from 8 to 15. The female parasites displayed a body length between 22.5 and 3.5 mm (mean: 2.95 mm), while males were smaller (1.5–2.0 mm, mean: 1.9 mm). Both sexes possessed four pairs of legs, a feature characteristic of caligid copepods. In females, the genital complex segment was evident, housing the oviduct channel, intestine, and immature eggs. The most conspicuous feature was the presence of long, bar-shaped egg sacs containing both mature and immature eggs, a defining characteristic of gravid female *C. clemensi*. The cephalothoracic shield was slightly oval-shaped, with a well-developed frontal plate adorned with diagnostic disk-shaped lunules in the cephalic region. Interestingly, the third leg-bearing segment was connected to the posterior portion of the cephalothorax via an apron-like tagma structure. The thorax comprised distinct segments leading into the genital complex segment**.** A clear differentiation was observed between the cephalothorax, further divided into cephalic, lateral, and thoracic zones, and the abdomen with its posterior tagma, including the abdomen and caudal rami, which was notably larger than the thoracic zone. These detailed morphological characteristics confirmed the identification of *C. clemensi* as the causative agent of the observed mortality in seabass and mullet populations (Figs. 2, 3).

### Molecular identification of *Caligus* sp.

In this study, a 634 bp fragment of the cytochrome oxidase I (COI) gene was sequenced from the identified *Caligus* sp., with the sequences deposited in GenBank under accession numbers PP549199 and PP549200. Intriguingly, the two sequences derived from distinct seabass and mullet species exhibited identical *COI* regions, indicating the conservation of this gene segment across *C. clemensi* populations. Comparative analysis unveiled a high similarity between the obtained sequence (PP549199) and known sequences, particularly with *C. clemensi* (HQ157566, HM582236, AM235887), showing a similarity range of 99.84–99.68%. This firmly establishes the species designation as *C. clemensi*. Further comparisons indicated decreasing identities, ranging from 87.67 to 82.52% with other *Caligus* species, such as *C. rotundigenitalis*, *C. quadratus*, *C. bonito*, *C. mutabilis*, *C. rogercresseyi*, *C. elongates* and *C. aesopus*. Additionally, a similarity range of 82.28–80.20% was observed when comparing the sequence with *Lepeophtheirus* spp. isolates, including *L. salmonis*, *L. frecuens*, and *L. confusum*.

**Fig. 4:**
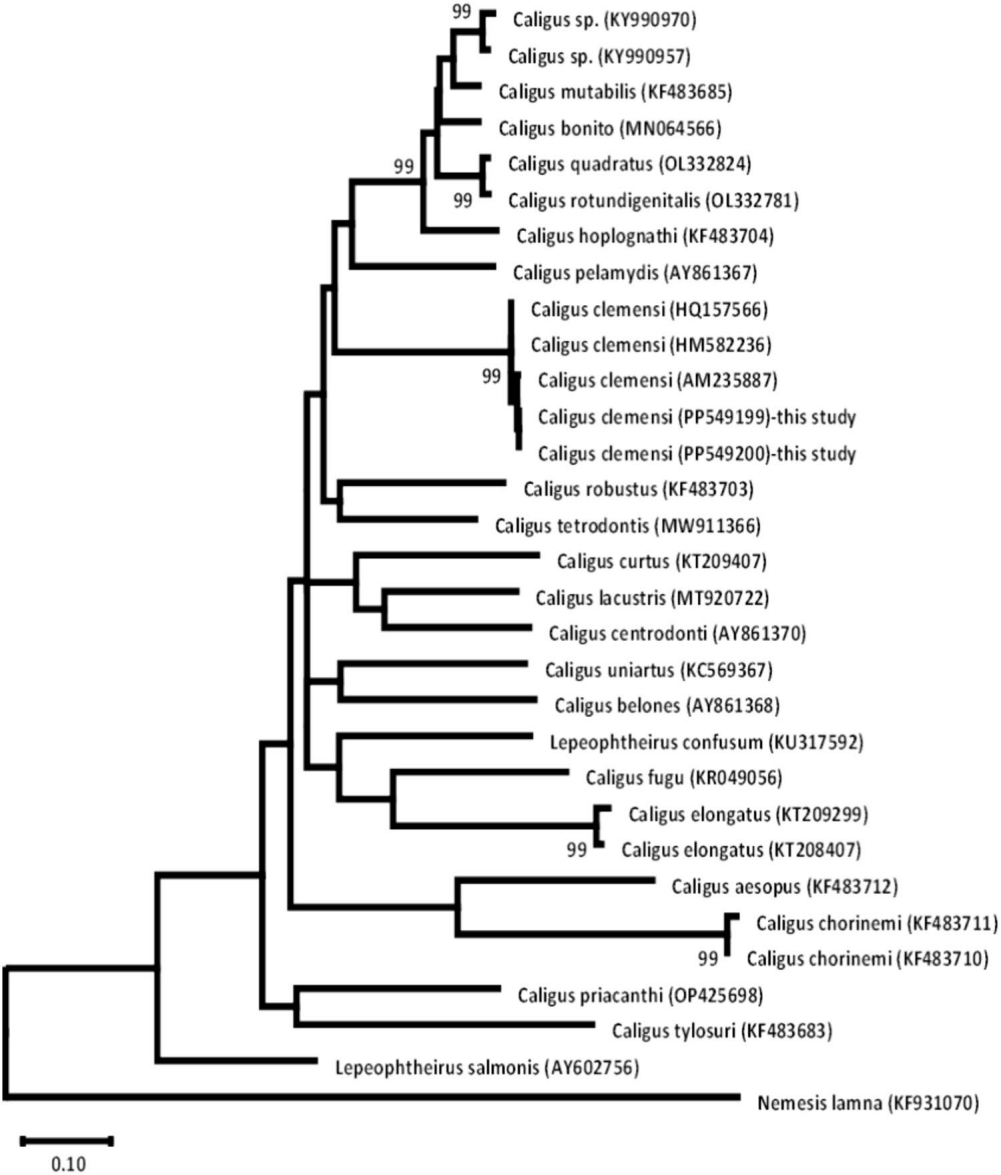
Phylogenetic tree constructed using the maximum composite likelihood model based on *COI* sequences. The analysis included the present *C. clemensi* (PP549199, PP549200) and other closely related members of the Caligidae family retrieved from GenBank.

The resulting phylogenetic tree (Fig. 4) showed that the two *C. clemensi* sequences (PP549199 and PP549200) obtained in this study form a strong monophyletic clade with three previously reported *C. clemensi* sequences (HQ157566, HM582236, and AM235887), supported by a bootstrap value of 99%. Within the *C. clemensi* clade, the isolates from this study (PF549199 and PF549200) form a distinct subgroup, suggesting their potential genetic variation. The *C. clemensi* clade is closely related to other important sea lice species, such as *C. robustus*, *C. tetrodontis*, *C. bonito, C. quadratus*, and *C. mutabilis*. More distantly related *Caligus* spp, such as *C. curtus*, *C. lacustris*, *C. centrodonti*, *C. aesopus*, *C. belones*, *C. elongatus* and *Lepeophtheirus salmonis* (a closely related sea lice genus), form separate clades, reflecting their genetic divergence from the *C. clemensi* isolates and other species within the larger group. Notably, the *C. clemensi* clade nested within a larger monophyletic *Caligus* group, distinct from other copepods genera like *Lepeophtheirus* (represented by *L. salmonis* and *L. confusum*) and the outgroup genus Nemesis (*N. lamna*). This phylogenetic analysis confirmed the morphological identification and provided insights into the taxonomic classifications of *C. clemensi* within the family Caligidae.

### Bacteriological examination

Eight *Vibrio* isolates were recovered from infected seabass and mullet. Gram staining revealed gram-negative, curved rods in all isolates. On TCBS agar, seabass isolates formed larger yellow colonies (2–4 mm), while mullet isolates produced smaller yellow colonies (2–3 mm). The dominant colony type from each fish species was purified and subjected to further biochemical and molecular characterization. Biochemical tests confirmed that all isolates were oxidase and catalase-positive, sensitive to the vibriostatic agent O/129, and tolerant to up to 6% NaCl. API 20E definitively identified the isolates as *V. alginolyticus*.

### Molecular identification of bacteria

PCR successfully amplified the housekeeping gene *recA* from all eight *Vibrio* isolates, generating amplicons of 736 bp. Subsequent sequencing of these amplicons, followed by nucleotide BLAST analysis, confirmed the identity of all isolates as *V. alginolyticus*. The four *recA* sequences obtained from the European seabass exhibited 100% nucleotide identity and were deposited in the GenBank database under accession numbers PP554475–PP554478. Similarly, the four sequences from the mullet showed 100% nucleotide identity and were assigned accession numbers PP554479–PP554482. Comparative analysis between the two host groups revealed a high degree of sequence similarity (98.7%), with only seven nucleotide differences. This minor divergence suggested potential host-associated adaptations within the *V. alginolyticus* isolates.

The analysis of the current *recA* sequences revealed (99.73–99.07%) sequence similarities for *V. alginolyticus* type strains (FM204832T, KC954188T, CP006718T, and AJ842373T). When compared to other *Vibrio* spp, the sequence identities ranged from 96.18 to 93% for closely related species such as *V. harveyi*, *V. owensii*, *V. campbellii*, and *V. rotiferianus*, and from 92.94 to 85.75% for more distantly related species including *V. parahaemolyticus*, *V. mimicus*, *V. vulnificus*, and *V. cholerae*.

### Phylogenetic analysis of *V. alginolyticus*

**Fig. 5:**
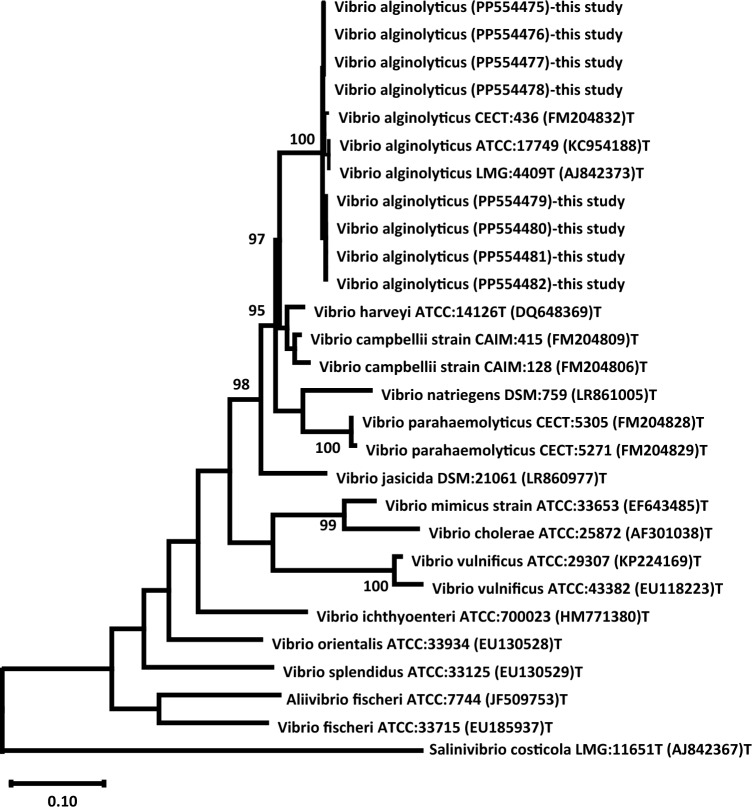
Phylogenetic tree based on partial *recA* genes showing distinct clusters among *V. alginolyticus* isolates (PP554475–PP554482). Related *Vibrio* species are included for comparison.

The maximum likelihood phylogenetic tree based on the *recA* gene sequences revealed a well-supported monophyletic clade comprising the eight *V. alginolyticus* isolates (PP554475–PP554482) from this study, with a high bootstrap value of 100% (Fig. 5). This *V. alginolyticus* clade formed a distinct lineage within the larger clade that encompassed other closely related *V. alginolyticus* typing strains, such as ATCC 17749, LMG 4409T, and CECT 436T, further confirming the species identification. Notably, the *V. alginolyticus* clade exhibited a close evolutionary relationship with other pathogenic *Vibrio* spp, including *V. harveyi, V. campbellii, V. natriegens, V. parahaemolyticus*, and *V. jasicida*, forming a well-supported monophyletic group with a bootstrap value of 98%. In contrast, more distantly related species like *V. mimicus*, *V. cholerae*, *V. vulnificus*, *V. ichthyoenteri*, *V. orientalis*, *V. splendidus*, *Aliivibrio fischeri*, and *Salinivibrio costicola* formed separate clades, reflecting their genetic divergence from the *V. alginolyticus* isolates. The phylogenetic analysis supported the accurate molecular identification of the isolates as *V. alginolyticus* and highlighted their close evolutionary relationship with other clinically relevant *Vibrio* spp within the broader genus.

### Antibiogram

The antibiotic susceptibility profiles of 8 *V. alginolyticus* isolates were determined against a panel of 8 antimicrobial agents encompassing 6 drug classes. All isolates (8/8, 100%) exhibited resistant phenotypes to the β-lactam antibiotics ampicillin, amoxicillin, and the aminoglycoside gentamicin. High rates of resistance were observed for the folate pathway inhibitor combination trimethoprim-sulfamethoxazole, with 5/8 isolates (62.5%) being resistant. Additionally, 4/8 isolates (50%) displayed resistance to the quinolone novobiocin. Moderate resistance frequencies of 37.5% (3/8 isolates) were seen against the tetracycline antibiotic oxytetracycline. In contrast, all isolates (8/8, 100%) were sensitive to the phenicol florfenicol.

### Cell-mediated immune response

**Fig. 6:**
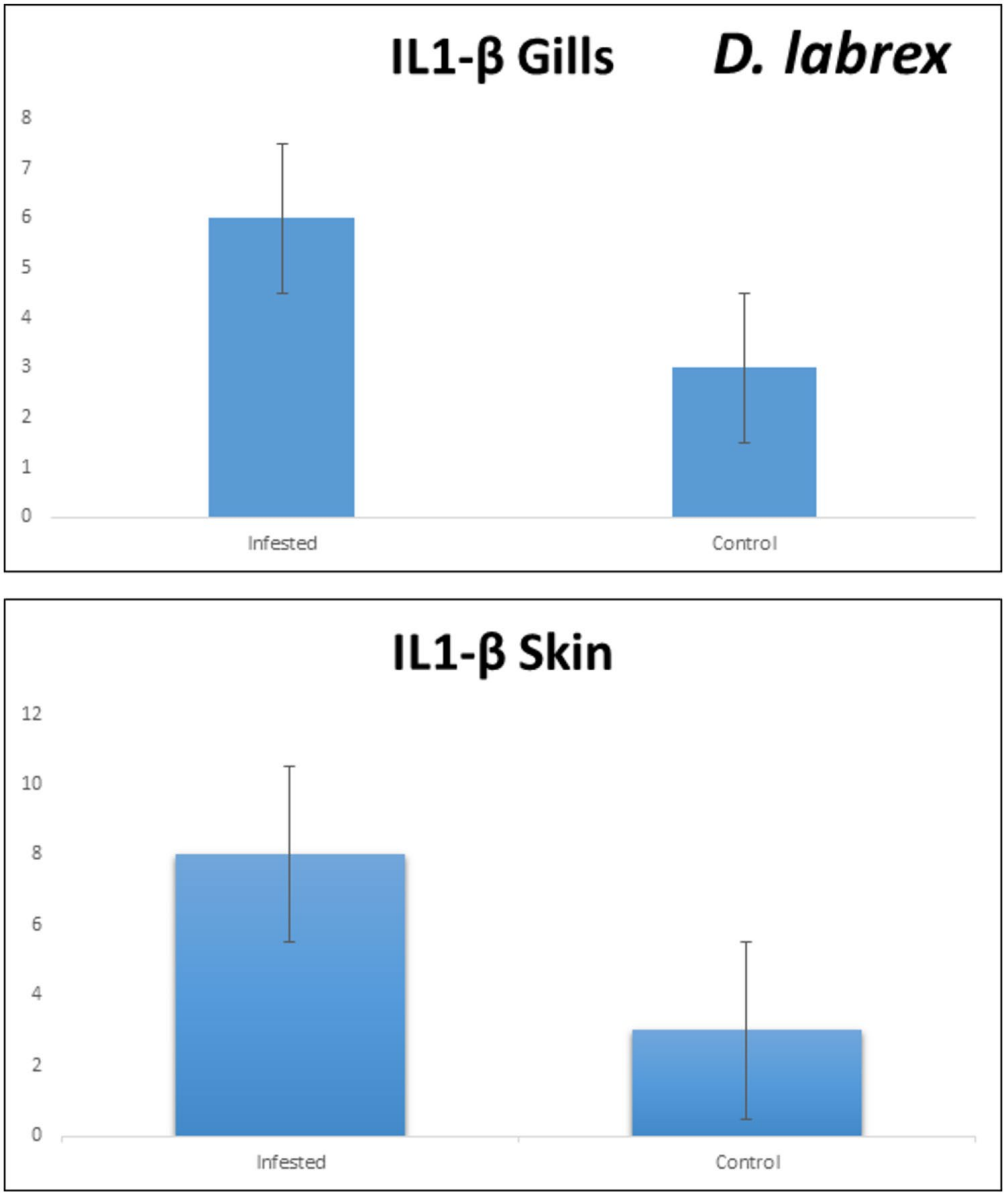
Transcript levels of *IL-1β* in gills and skin of European seabass.

**Fig. 7:**
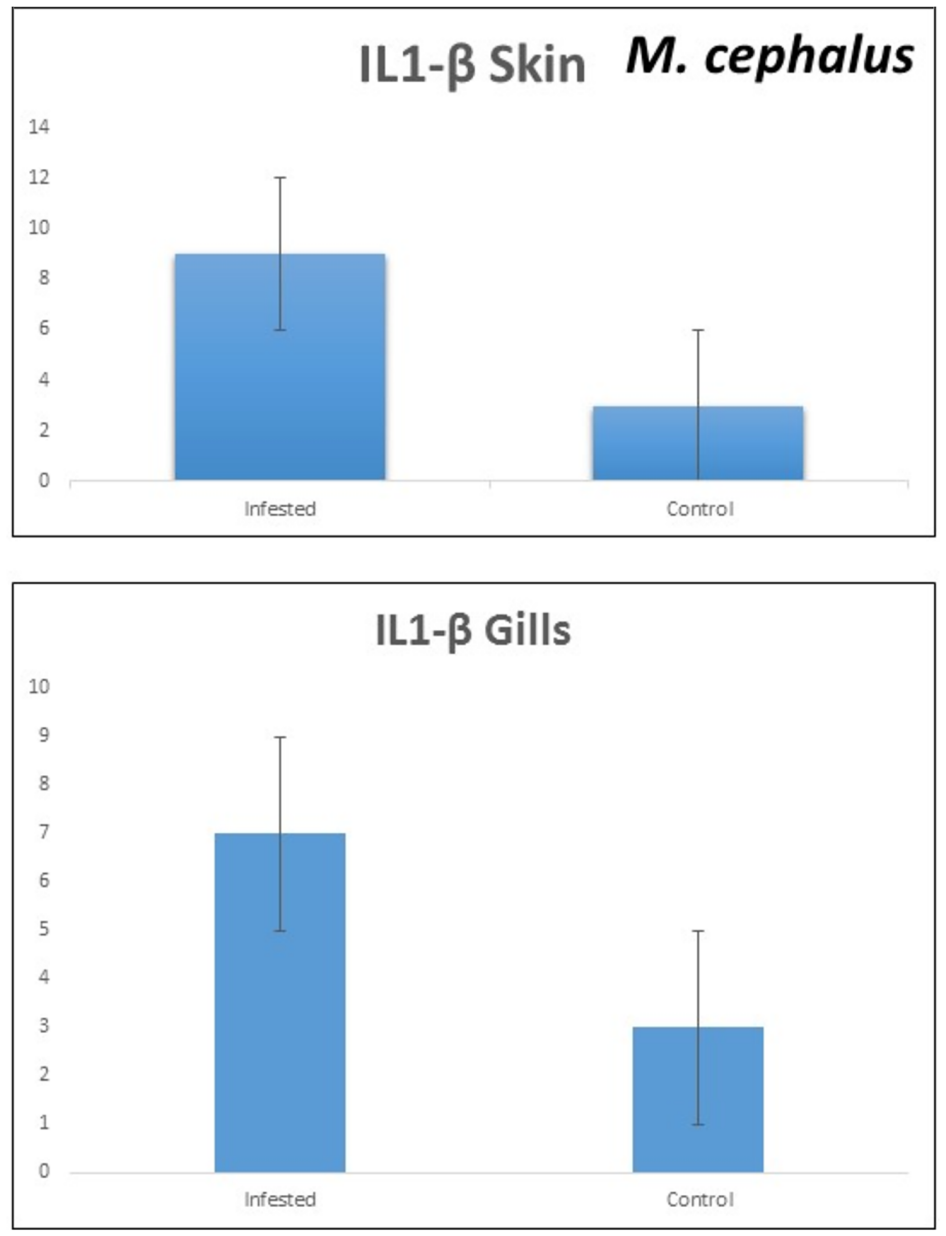
Transcript levels of *IL-1β* in gills and skin of grey mullet.

The expression of the inflammatory marker interleukin-1β (IL-1β) was significantly upregulated in both the gills and skin of infected European seabass (*D. labrax*) and mullet (*M. cephalus*) compared to non-infected control individuals. However, the gills exhibited a more pronounced increase in *IL-1β* transcript levels than the skin in both fish species. Specifically, in European seabass, *IL-1β* expression was markedly higher in the gills of infected fish, showing around a 15-fold upregulation relative to controls, while the elevation in skin samples was comparatively lower at approximately fivefold (Fig. 6). A similar pattern was observed in mullet, with infected individuals displaying a substantial 20-fold increase in *IL-1β* transcripts in the gills, and a moderate sevenfold increase in the skin compared to non-infected controls (Fig. 7). These findings indicate a robust cell-mediated immune response, particularly in the gills which are likely the primary site of pathogen entry and infection in these fish. The upregulation of the pro-inflammatory cytokine *IL-1β* suggests activation of the innate immune system as a defense mechanism against the invading pathogen.

### Histopathological examination

**Fig. 8:**
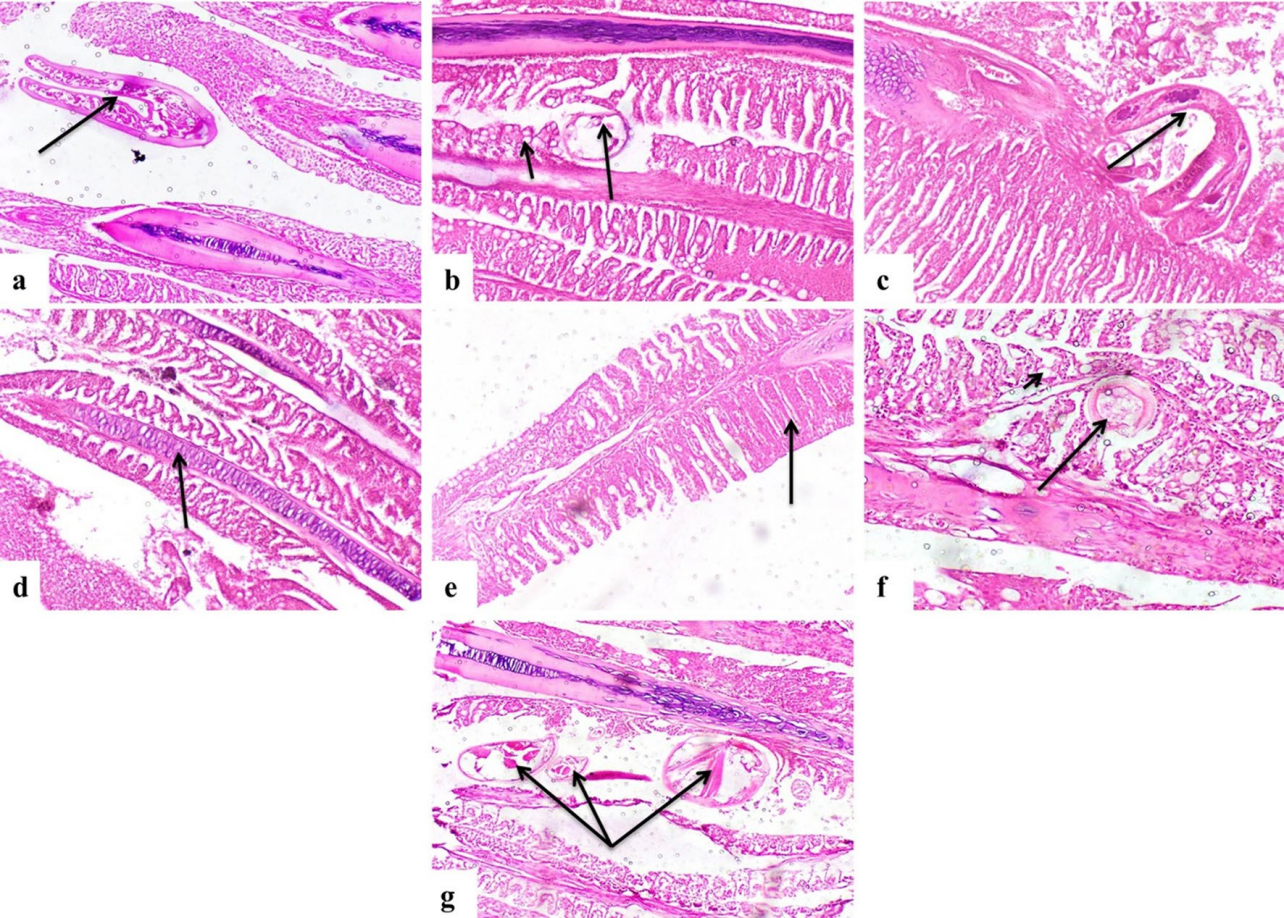
Photomicrograph of European seabass gills showing. (**a**) Presence of parasite between gills (arrow). (**b**) Parts of the parasite between gill lamellae (long arrow) with hyperplasia of mucus cells (short arrow). (**c**) The parasitic section between gills (arrow) with severe tissue reaction. (**d**) The proliferation of the cartilaginous core (arrow). (**e**) Hyperplasia and fusion of gill lamellae (arrow). (**f**) Presence of parasitic sections embedded in gills (long arrow) and activation of mucus cells (short arrow). (**g**) Some cases showing heavy parasitic infestation with severe tissue reaction (arrows).

**Fig. 9:**
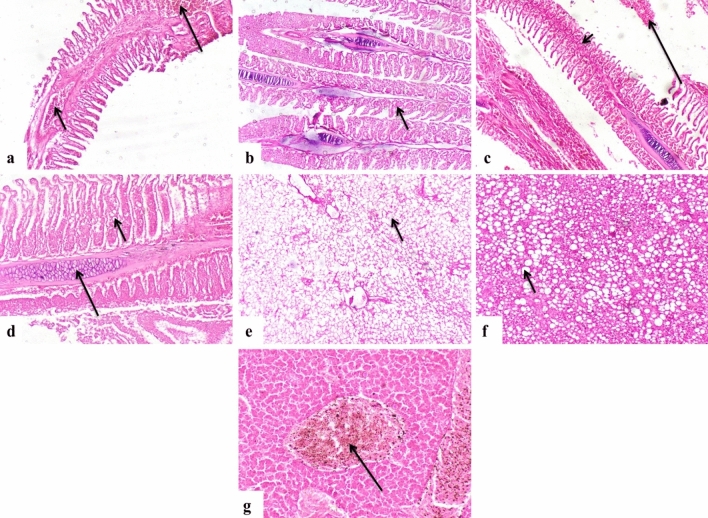
Photomicrograph of grey mullet. (**a**) Gills showing hemorrhage of gills (long arrow) and congestion of interstitial blood capillaries (short arrow). (**b**) Gills showing congestion of capillaries of gill lamellae (arrow). (**c**) Gills showing hemorrhage between gills (long arrow) and hyperplasia of primary gill lamellae (short arrow). (**d**) Gills showing the large number of mucus cells lining gills (short arrow) with hyperplasia of the cartilage core (long arrow). (**e**) Liver showing severe vacuolar degeneration of hepatocytes (arrow). (**f**) Liver showing widespread hepatocellular steatosis (arrow). (**g**) Liver showing severe congestion of the central vein (arrow).

Gills: Histopathological findings of the gills revealed the presence of parasites between the gills or even between the gill lamellae, accompanied by severe tissue destruction (Fig. 8a, b, and c). The proliferation of the cartilaginous core of the gills (Fig. 8d), hyperplasia, and fusion of secondary gill lamellae was also evident (Fig. 8e). Parasitic parts were observed embedded in the gills (Fig. 8f). Some infested fish exhibited heavy parasitic infestation with severe tissue reactions (Fig. 8g). The gills also showed lamellar hemorrhage and congestion of interstitial blood capillaries (Fig. 9a), congestion of lamellar capillaries (Fig. 9b), severe hemorrhage between the gills, and hyperplasia of primary gill lamellae (Fig. 9c). Infested gills displayed a large number of mucus cells lining the gills (Fig. 9d). Liver: The liver of infested fish exhibited severe vacuolar degeneration of hepatocytes (Fig. 9e). Some cases showed hepatocellular steatosis and a considerable number of affected hepatocytes (Fig. 9f). The liver also exhibited severe congestion of hepatic blood vessels (Fig. 9g).

### Field treatment trial

A field trial evaluated the efficacy of the 7-day treatment regimen for *C. clemensi* and *V. alginolyticus* co-infections in European seabass and grey mullet. The regimen comprised H_2_O_2_ immersion baths, Lice-Less feed incorporation, and in-pond supplementation with Sanolife Pro-W probiotics. Pre-treatment in March, seabass exhibited 65% prevalence of *C. clemensi*, with most infected fish showing co-infection with *V. alginolyticus* (60 out of 65). Similarly, mullet had a 40% prevalence, with all infested fish co-infected. The parasitic intensity was high, with seabass showing 8.2 ± 2.1 and 9.4 ± 1.6 parasites on average on gills and skin respectively, while mullet had 5.1 ± 0.2 and 7.5 ± 2.3 (Table 1).

Post-treatment by mid-April, examinations showed a significant reduction of *C. clemensi* in treated groups. Seabass prevalence dropped from 65 to 10%, with drastically reduced intensity to an average of 2.1 ± 1.3 on gills and 1.3 ± 0.2 on skin. In treated mullet, prevalence reduced from 40 to 5%, with intensity dropped to an average of 1.2 ± 0.1 on gills and 1.1 ± 0.3 on skin. The co-infection rates also decreased substantially, with only 6 out of 10 infested seabass and 2 out of 5 infested mullet showing *V. alginolyticus* co-infection. By mid-April, most fish appeared clinically healthy, with a residual mortality rate of only 0.5%. No discernible adverse effects were observed and fish activity and feeding patterns improved post-therapy. By the end of May 2022, no morbidity or mortality cases were recorded in treated groups.

Contrastingly, the two untreated control ponds showed persistent high infestation rates throughout the study period (68% seabass, 44% mullet), with all infested fish co-infected with *V. alginolyticus*. The parasitic intensity remained high in both fish species, with seabass showing 9.7 ± 1.1 on gills and 10.2 ± 1.4 on skin, and mullet exhibiting 6.3 ± 2.1 on gills and 8.4 ± 1.7 on skin. Furthermore, control groups exhibited no signs of improvement. By mid-April, mortality reached 30% in seabass and 20% in mullet in untreated groups, with retrieving *V. alginolyticus* from all infested fish confirming uncontrolled secondary bacterial infections. This high mortality rate, coupled with the persistent high infestation and intensity rates, underscores the severe impact of untreated *C. clemensi* and *V. alginolyticus* co-infections on fish health and survival.

## Discussion

The present study provides comprehensive insights into a significant disease outbreak characterized by *C. clemensi* infestations and subsequent *V. alginolyticus* infections in European seabass (*D. labrax*) and flathead grey mullet (*M. cephalus*) farmed in the Deeba Triangle region of Egypt. Our findings revealed a complex interplay between parasite, bacteria, and host, with significant implications for mariculture management in this dynamic environmental setting^4^. The co-occurrence of *C. clemensi* and *V. alginolyticus* in this outbreak aligns with a growing trend of multi-pathogen infections observed in aquaculture systems worldwide. This phenomenon, often referred to as polymicrobial disease, is increasingly recognized as a significant challenge in marine fish farming^1,8,9^. The synergistic effects of parasitic and bacterial pathogens can lead to more severe disease outcomes and complicate management strategies. Our findings contribute to the growing body of evidence supporting the importance of considering multiple pathogens in aquaculture disease management. The prevalence and intensity of *Caligus* infestation observed were remarkably high, showing a rapid increase over time. In seabass, prevalence rose from 25% in February to 65% in March, and further to 68% in untreated groups by April. Similarly, in mullet, prevalence increased from 15% in February to 40% in March, reaching 44% in untreated groups by April. This rapid escalation underscores the aggressive nature of these parasites in aquaculture settings and highlights a critical window for intervention, consistent with previous reports of severe caligid infestations in mariculture^8^.

The accurate identification of the caligid species involved in the present outbreak was facilitated through a combination of morphological and molecular techniques. The morphological examination, employing taxonomic keys and criteria such as antennae segmentation, setae counts, and egg sac measurements^21,22^, initially suggested the involvement of a *Caligus* sp. However, the limitations of morphological identification within this genus, due to overlapping characteristics, necessitated the use of molecular approaches to establish a definitive taxonomic classification. Sequencing and phylogenetic analysis of the *COI* gene, a widely employed molecular marker for species identification and delineation in copepods^39^, confirmed the identity of the caligid species as *C. clemensi*. The maximum likelihood analysis of the *COI* sequences robustly clustered the obtained sequences with other sequences of *C. clemensi*, further confirming the taxonomic identity. The incorporation of molecular techniques has proven instrumental in resolving the taxonomic ambiguities often encountered in the identification of caligid copepods based solely on morphological traits^10^.

The co-occurrence of *V. alginolyticus* in the infested fish samples further underscored the complexity of this disease outbreak. *V. alginolyticus* is a ubiquitous marine bacterium known to cause vibriosis, a significant disease in aquaculture operations worldwide^16^. The molecular identification of the bacterial isolates, based on the *recA* gene sequence analysis, unequivocally confirmed their identity as *V. alginolyticus*. The *recA* gene, encoding a protein involved in DNA recombination and repair, has been widely used for the accurate identification and phylogenetic analysis of *Vibrio* spp.^40,41^. The co-infection dynamics observed in this study highlight the potential for caligid copepods to serve as vectors for the transmission of bacterial pathogens, including *V. alginolyticus*. Previous studies have detected the presence of *V. alginolyticus* within specific *Caligus* spp., such as *C. lalandei* and *C. minimus*^16^, supporting the hypothesis that these parasites may facilitate the dissemination of vibriosis among fish populations.

The histopathological examination provided valuable insights into the pathological changes induced by the dual infection. The gills exhibited severe tissue damage, hyperplasia, and hemorrhage, reflecting the direct impact of *C. clemensi* and the subsequent bacterial invasion. These findings are consistent with previous studies on caligid copepod infestations and associated secondary bacterial infections^8,9^. The liver pathology, including vacuolar degeneration, steatosis, and vascular congestion, further underscored the systemic effects of the disease complex on the fish hosts.

Interestingly, the histopathological observations correlated strongly with the observed immune response, particularly the upregulation of *IL-1β* in both the gills and skin of the infected fish. This significant increase in *IL-1β* expression indicated a robust cell-mediated immune response against the invading pathogens, aligning with previous studies reporting the pivotal role of *IL-1β* in fish immunity during parasitic and bacterial infections^30,31^. *IL-1β* plays a crucial role in the innate immune response by mediating inflammatory processes and regulating the expression of other cytokines and immune effector molecules^42^. The pronounced increase in *IL-1β* expression in the gills, the primary site of pathogen entry, suggested a localized heightened inflammatory reaction to combat the disease. This correlation between tissue damage and immune response highlights the complex host–pathogen interactions at play in this co-infection scenario.

The antimicrobial resistance (AMR) observed in the *V. alginolyticus* isolates is particularly concerning and reflects a broader trend in aquaculture environments globally. In this study, the *V. alginolyticus* isolates exhibited extensive resistance to most of the assessed antibiotics, a finding consistent with the alarming trend in aquaculture environments. All isolates (100%) showed resistance to β-lactams (ampicillin and amoxicillin) and the aminoglycoside gentamicin, indicating a critical loss of efficacy in these commonly used drug classes. Furthermore, high resistance rates to trimethoprim-sulfamethoxazole (62.5%) and novobiocin (50%) suggested that folate pathway inhibitors and quinolones are also becoming less effective. Even oxytetracycline showed reduced effectiveness with 37.5% of isolates being resistant. The high resistance levels observed across multiple drug classes severely limit treatment options, potentially leading to therapeutic failures and economic losses in aquaculture^43^. This widespread AMR poses a significant threat not only to fish health and aquaculture productivity but also to human health through the potential transfer of resistant genes to human pathogens^44^.

This widespread antibiotic resistance in *V. alginolyticus* underscores the urgency to adopt alternative control strategies that minimize antimicrobial use. A paradigm shift towards preventive measures and alternative therapies is critical to manage this growing threat and ensure aquaculture sustainability^44,45^. The alarming AMR situation in aquaculture necessitates a shift towards more sustainable disease management practices.

In response to these challenges, we implemented an integrated treatment protocol that effectively eliminated *C. clemensi* infestations and controlled secondary bacterial infections in the field trial. This comprehensive approach, comprising physical (hydrogen peroxide immersion baths), chemical (nature-identical compounds), and biological (probiotics) control measures, aligns with emerging strategies for sustainable aquaculture disease management^18^. The use of hydrogen peroxide as an initial disinfectant was informed by its broad-spectrum activity against both ectoparasites and bacterial pathogens^46^. The proposed action mechanism for hydrogen peroxide involves the production of bubbles in the copepod hemolymph, leading to mechanical paralysis and detachment from the host^47^.

The nature-identical compounds (NICs) used in this study have demonstrated promising antiparasitic, antimicrobial, and immunomodulatory properties in other studies^18,48–51^. NICs are chemically synthesized to mimic bioactive components of plant-derived essential oils and oleoresins^48^. The selected blend, containing phenolic monoterpenoids (thymol and carvacrol) and aromatic aldehyde monoterpenes (cinnamaldehyde), has demonstrated promising efficacy against sea lice in Atlantic salmon due to its ability to alter stress and signalling regulators related to epidermal mucus proteins^48,52^. The blend demonstrated antimicrobial activity against *Vibrio* spp., known for their antibiotic resistance^18^. By disrupting bacterial membranes, inhibiting quorum sensing, and preventing biofilm formation, it offers a potential solution to this challenge. Additionally, its immunomodulatory properties, including cytokine regulation in seabass^53^, suggest a broader approach to disease control by enhancing host resilience.

The incorporation of probiotics aimed at restoring gut microbiome balance and enhancing innate immune defenses against secondary bacterial infections^38^. This decision was guided by antibiogram results, highlighting the need for alternatives to conventional antibiotics. Previous studies have shown that probiotics can effectively control diseases by competitively excluding pathogenic bacteria^38,54^. The high prevalence of *V. alginolyticus* in control groups, contrasted with its absence in treated fish, suggested that the probiotic intervention was instrumental in controlling secondary bacterial infections.

The efficacy of this integrated approach in our field trial, evidenced by the significant reduction in parasite prevalence and the absence of *V. alginolyticus* in treated groups, supports the viability of non-antibiotic disease management strategies in aquaculture. The stark contrast in infection rates and mortality between treated and untreated populations underscores the potential of such integrated protocols in managing complex disease outbreaks. However, it is important to note that while our study demonstrates the effectiveness of this particular combination of treatments, further study is needed to optimize these protocols and explore other potential natural compounds and probiotics for aquaculture disease management. The development of standardized, evidence-based protocols for integrated disease management in aquaculture should be a priority for future studies.

While dual infection challenge experiments are ideal for confirming co-infection dynamics, they were not feasible in this commercial setting due to the risks of outbreaks. Instead, this study leveraged a natural outbreak as a real-world model. The pronounced health disparities between treated and untreated groups, along with pathogen identification coupled with molecular and histopathological findings, strongly support the *C. clemensi-V. alginolyticus* co-infection hypothesis. Furthermore, these observations align with previous controlled studies on fish parasite-bacteria co-infections. In Atlantic salmon, *C. rogercresseyi* infestation significantly increased susceptibility to *Piscirickettsia salmonis*^55^. Similarly, *Lepeophtheirus salmonis* was shown to enhance *Aeromonas salmonicida* infections^56^. These studies, conducted under controlled conditions, provide additional support for the co-infection dynamics observed in this field setting.

In conclusion, this study elucidated a severe co-infection of *C. clemensi* and *V. alginolyticus* in Egyptian seabass and mullet mariculture. The complex interplay between parasitic infestation, bacterial infection, host immune response, and tissue pathology underscores the multifaceted nature of disease outbreaks in aquaculture settings. The high antibiotic resistance observed in *V. alginolyticus* isolates highlights the urgent need for alternative disease management strategies in aquaculture. Our integrated treatment protocol, combining physical, chemical, and biological control measures, offers a promising approach to controlling such complex disease outbreaks while minimizing reliance on antibiotics. These findings contribute to our understanding of polymicrobial diseases in aquaculture and provide valuable insights for developing sustainable disease management strategies in marine fish farming operations. Future research should focus on optimizing these integrated protocols and exploring other potential natural compounds and probiotics for aquaculture disease management, with the goal of developing standardized, evidence-based protocols for integrated disease management in aquaculture.

## Data Availability

All data that support the findings of this study are available upon request from the corresponding author.
